# Carburization Kinetics of Zircalloy-4 and Its Implication for Small Modular Reactor Performance

**DOI:** 10.3390/ma15228008

**Published:** 2022-11-12

**Authors:** Erofili Kardoulaki, Najeb Abdul-Jabbar, Darrin Byler, Md Mehadi Hassan, Shane Mann, Tim Coons, Josh White

**Affiliations:** Los Alamos National Laboratory, P.O. Box 1663, Los Alamos, NM 87545, USA

**Keywords:** small modular reactors, zircaloy, carburization, kinetics, indentation

## Abstract

Carburization of cladding materials has long been a concern for the nuclear industry and has led to the restricted use of high-thermal conductivity fuels such as uranium carbides. With the rise of small modular reactors (SMRs) that frequently implement a graphite core-block, carburization of reactor components is once more in the foreground as a potential failure mechanism. To ensure commercial viability for SMRs, neutron-friendly cladding materials such as Zr-based alloys are required. In this work, the carburization kinetics of Zircaloy-4 (Zry-4), for the temperature range 1073–1673 K (covering typical operating temperatures and off-normal scenarios) are established. The following Arrhenius relationship for the parabolic constant describing ZrC growth is derived: K_p_ (in μm^2^/s) = 609.35 exp(−1.505 × 10^5^/RT)). Overall, the ZrC growth is sluggish below 1473 K which is within the operational temperature range of SMRs. In all cases the ZrC that forms from solid state reaction is hypo-stoichiometric, as confirmed through XRD. The hardness and elastic modulus of carburized Zry-4 are also examined and it is shown that despite the formation of a ZrC layer, C ingress in the Zry-4 bulk does not impact the mechanical response after carburization at 1073 K and 1473 K for 96 h.

## 1. Introduction

Small modular reactors (SMRs) are currently under development by multiple government agencies and commercial entities to support terrestrial power applications where grid support is limited. SMRs present unique design and materials challenges and must be commercially attractive in order to succeed. For example, a high thermal conductivity throughout the reactor’s lifetime and the implementation of a robust fuel-cladding system, to retain fission products during normal and off-normal operation, are key. Therefore, fuel-cladding assemblies must be optimized to promote heat transfer, mechanical integrity, and fission gas retention. 

For many SMR designs under consideration, a graphite core-block is proposed [1,2]. This introduces the question of carburization and carbon corrosion of the cladding materials. Carburization of austenitic steel cladding from carbide fuel or from C impurities present in nitride fuel, used in liquid-metal fast breeder reactors, for example, was previously found to be a large concern due to embrittlement [3,4,5]. At temperatures higher than in light water reactors (LWRs), at which SMRs are expected to operate (e.g., 873 K for LWRs versus 1273 K for SMRs), the thermomechanical impact of carburization is unexplored for many materials. This includes examinations of the carburization kinetics and extent of carbon corrosion as well as the impact of these reactions on the thermal conductivity and mechanical integrity of the substrate materials. An example of such a material that has not been previously studied with regards to carburization and its impacts on performance are the well-studied Zr-based alloys which have been used for multiple decades as fuel cladding in LWRs. 

Zr-based alloys have many advantageous properties, including favorable neutronics, good fission gas retention, and an established track record of performance in LWRs [6,7]. In recent years the nuclear industry has sought to find alternatives to Zr-based LWR cladding systems as steam oxidation of Zr at high temperatures (>1273 K) leads to the generation of excessive heat and hydrogen gas with detrimental effects to a potential ongoing accident. The driving force for the replacement of Zr cladding, however, does not apply to SMR operational conditions since in many designs water is not used as a reactor coolant [1,2]. The search for a Zr replacement due to water/stream interactions has led the LWR industry to explore alternative cladding materials, with Cr-coated Zr-based alloys and advanced stainless steel alloys (i.e., FeCrAl) emerging as realistic near-term replacements [8]. Other options such as silicon carbide claddings are seen as longer-term alternatives that could provide significant benefits, with similar or better neutronic efficiency to Zr, if manufacturing obstacles are addressed [9]. 

Substitution of Zr-based alloys for Fe ones achieves increased corrosion resistance in steam/water environments, but it does come with a neutronic penalty. This means that fuels with higher U densities need to be implemented instead of traditional UO_2_ to achieve similar neutronic performance as that of the UO_2_-Zr system. For this reason, uranium silicides have been previously explored by Westinghouse Electric and more recently uranium mononitride [10,11]. It is obvious then that when deciding on a cladding substitution for a reactor system, the fuel is also a crucial component that cannot be ignored. These high U-density fuels can provide many benefits; however, they do come with associated operational costs that need to be considered when assessing commercial viability. Some of the key thermomechanical properties for Zry-4 and for a prominent FeCrAl candidate (Kanthal APMT) are summarized in Table 1, showcasing the advantages of Zr-based alloys. For the purposes of this work, which is targeted at SMR systems that do not use water as a coolant, it is clear that Zr-based alloys can provide many benefits if reactions with the graphite core-block are manageable under relevant operational conditions. Therefore, understanding and controlling the potentially deleterious interface reactions between the fuel, cladding, and the graphite core-block is very important for the success of SMRs. Unforeseen thermochemical interactions between the system constituents could jeopardize the mechanical stability and heat transfer of the system, which are important for their effective commercial viability. 

In this work, high temperature interactions at the Zircaloy-4 (Zry-4) and C interfaces are examined from 1073–1673 K. The selected temperature range covers typical SMR operating temperatures (1073–1173 K) as well as higher temperatures to accelerate kinetics and simulate off-normal reactor conditions. The kinetics of ZrC growth on Zry-4 are ascertained, its effects on cladding mechanical properties are measured, and the results’ technological implications on the design of SMRs are discussed.

## 2. Materials and Methods

Diffusion couple experiments between Zry-4 and graphite were performed at four different temperatures to establish the temperature-dependent ZrC growth kinetics as a function of time. Each couple consisted of a Zry-4 sample, a graphite sample, and a tungsten (W) weight that provided pressure (~100 kPa) to ensure contact between the constituent samples. The selected temperatures for the diffusion couple testing were 1673 K, 1473 K, 1273 K, and 1073 K. Different time durations were tested ranging from 12 to 96 h for select temperatures. A solid-state pack carburization process was also used to convert the surface of a Zr substrate (ATI, Zr Cystal Bar) to ZrC. Pack carburization was selected as a comparison method to a solid-state reaction since it is a technique that can be scaled up more readily. An assessment of pack carburization then would be important if it was found that a thin layer of ZrC is advantageous at the start of the reactor’s lifetime. In this case, graphite powder (Alfa Aesar, Ward Hill, MA, USA, 99.9995%) was placed into a graphite crucible to form a powder bed for the Zr sample. Graphite powder was packed around the rest of the Zr substrate to completely encompass the substrate. The crucibles were then heated to 1623 K for three different isothermal holds; 6 h, 12 h, and 18 h. All testing took place under gettered ultra-high purity argon flowing at 500 mL/min. Following the carburization tests, each sample was cross-sectioned, mounted in epoxy, and polished for microstructural characterization. The thickness of the ZrC coating that was developed was characterized using an optical microscope (OLYMPUS) and an FEI Apreo SEM equipped with an energy-dispersive spectral analyzer (EDS). X-ray diffraction (XRD) was also performed to assess the ZrC growth. These measurements were performed using a Bruker D2 diffractometer (D2 Phaser, Bruker Corp., Billerica, MA, USA) using Cu Kα radiation from 10–90° 2θ with a step size of 0.02°.

Room temperature nanoindentation was carried out to assess any changes in the hardness and modulus of the Zry-4 substrate material as a result of C diffusion from the graphite or the ZrC layer. The samples selected for the nanoindentation were those that were exposed to graphite for the longest time (i.e., 96 h) at 1073 K and 1473 K. The results were compared to data for an as-received Zry-4 sample. An I-Micro tester was used to perform continuous stiffness measurements (CSM) using a 4 × 4 grid indent separated by 50 μm [19,20]. A diamond Berkovich triangular indenter was used for its high hardness and elastic modulus to minimize the contribution of the indenter itself to the measured displacement. The hardness and the elastic modulus were calculated from the load-displacement curves [21].

## 3. Results

**Figure 1:**
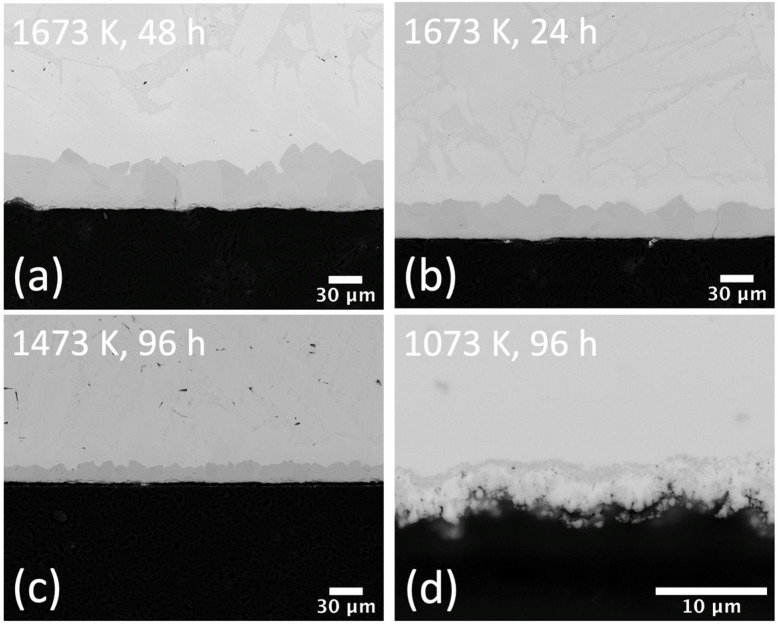
SEM images of carburized Zr samples through solid state reaction at (**a**) 1673 K for 48 h (**b**) 1673 K for 24 h, (**c**) 1473 K for 96 h, and (**d**) 1073 K for 96 h.

**Figure 2:**
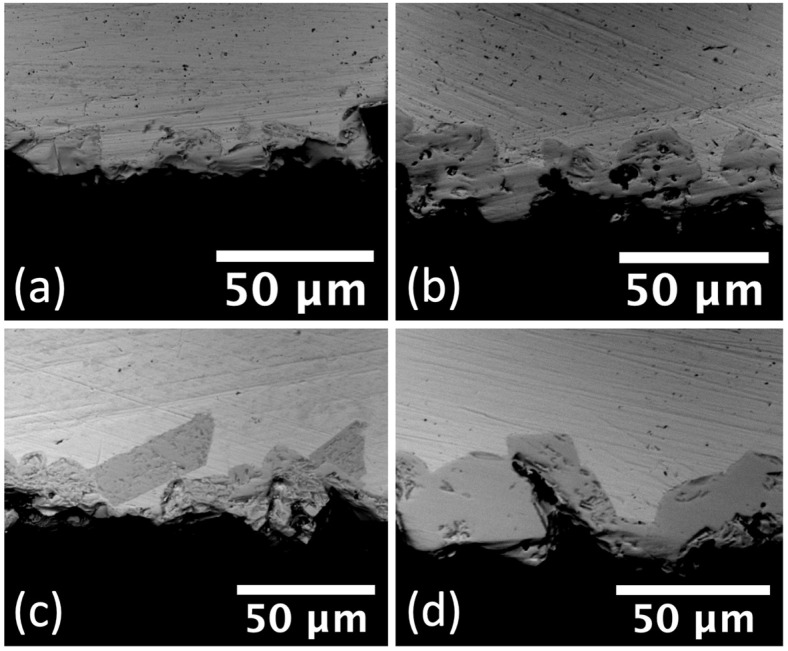
SEM images of carburized Zr samples through pack carburization at 1623 K for (**a**) 6 h, (**b**,**c**) 12 h, and (**d**) 18 h.

In Figure 1, SEM images of the resulting ZrC layers that grew from solid state reaction at various conditions are shown. In all cases the ZrC was dense and the thickness grew with increases in temperature and time exposure. The temperature was shown to be a more impactful parameter than time for the in-situ growth of ZrC. For example, the average ZrC thickness for a reaction at 1623 K for 48 h was 40 μm, whilst at the same temperature after exposure at 24 h the resulting ZrC thickness was 30 μm. For temperatures below 1473 K, the ZrC thickness was small even when implementing long exposures of 96 h. For example, the average ZrC thickness for 96 h exposures at 1473 K and 1073 K was 17 μm and 3 μm, respectively. In Figure 2, SEM images for the ZrC layers that grew through pack carburization at 1623 K after different time exposures are shown. Again, as time exposure increases, so does the ZrC thickness. Albeit, this effect is subtle for the chosen time exposures. Here the ZrC thickness increases approximately from 13 μm, to 19 μm, to 26 μm for exposure time increases from 6 to 12 to 18 h, respectively. Whilst these average ZrC thickness values are higher compared to those of layers grown through solid-state reaction, this is mostly due to the irregular nature of the layers grown under pack carburization (see Figure 2). Aside from the large variations in thickness, the ZrC layers of Figure 2 also appear to have diminished in quality compared to the ones of Figure 1, with many pores and considerable cracking. 

**Figure 3:**
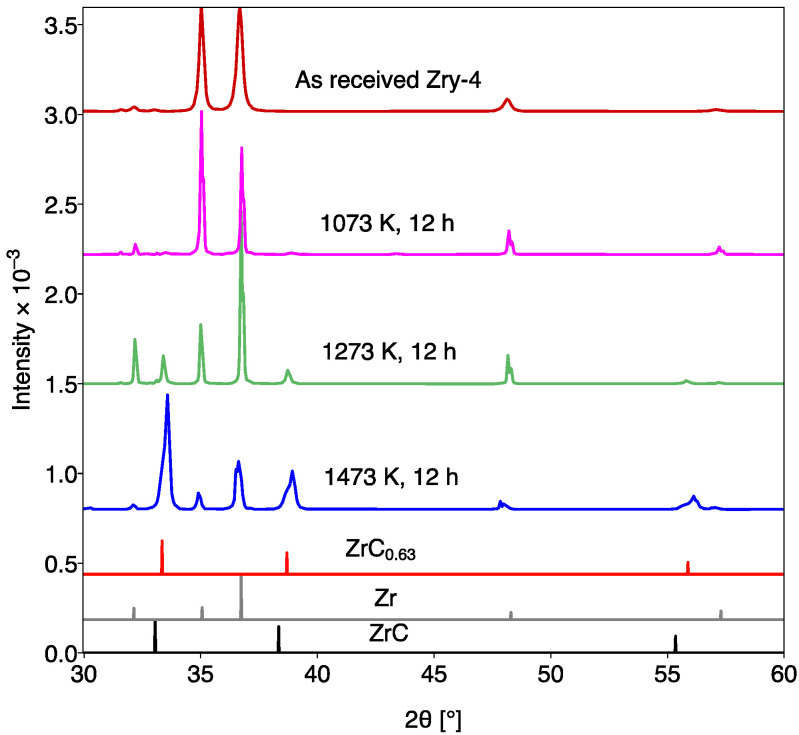
XRD patterns of as received Zry-4 (solid sample) and carburized Zry-4 (ZrC layer grown on solid Zry-4 samples) at 1073 K, 1273 K, and 1473 K for 12 h. Reference patterns for ZrC_0.63_ (ICSD 14822 [22]), ZrC (ICSD 159874 [23]), and Zr (ICSD 53785 [24]) are also shown.

In Figure 3 the XRD patterns from Zry-4 samples carburized for 12 h at 1073 K, 1273 K, and 1473 K, are shown compared to as-received Zry-4. Additionally, reference peaks corresponding to Zr metal, stoichiometric ZrC, and hypo-stoichiometric ZrC_0.63_ are included to serve as a comparison with the carburized Zry-4 specimens. The results suggest that the formation of ZrC predominantly occurs at elevated temperatures (1273 K and 1473 K). At 1073 K, ZrC diffraction peaks are absent, which indicates minimal carburization of Zry-4 at lower temperature regimes after 12 h. SEM images corroborate the XRD results, where only sparse ZrC layers (~1 μm in thickness) are observed in the specimen treated at 1073 K for 12 h, whilst only ~3 μm of ZrC form after 96 h (see Figure 1d). 

**Figure 4:**
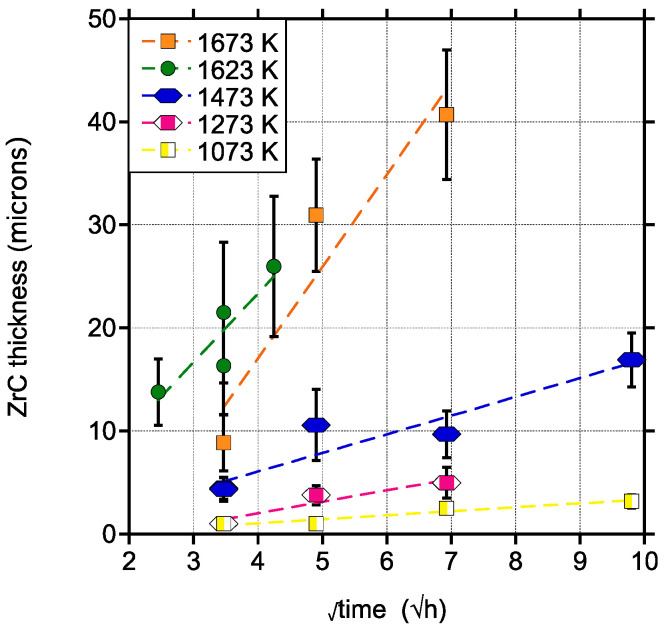
ZrC layer thickness versus the square root of time during the isothermal holds from solid state carburization (1073, 1273, 1473, 1673 K) and pack carburization (1623 K).

In order to fully assess the kinetics of ZrC layer growth, a variety of temperatures and times were tested and the obtained ZrC thicknesses, as determined from microstructural examinations (some of which are presented in Figure 1 and Figure 2), are shown in Figure 4. Each data point in Figure 4 represents the average from measurements taken from at least 3 images and over 50 measurements. In all cases a parabolic rate law relationship is observed [25]:

(1) $$Xc_{\;}=\sqrt{\left(K_{p}t\right)}+A$$

**Figure 5:**
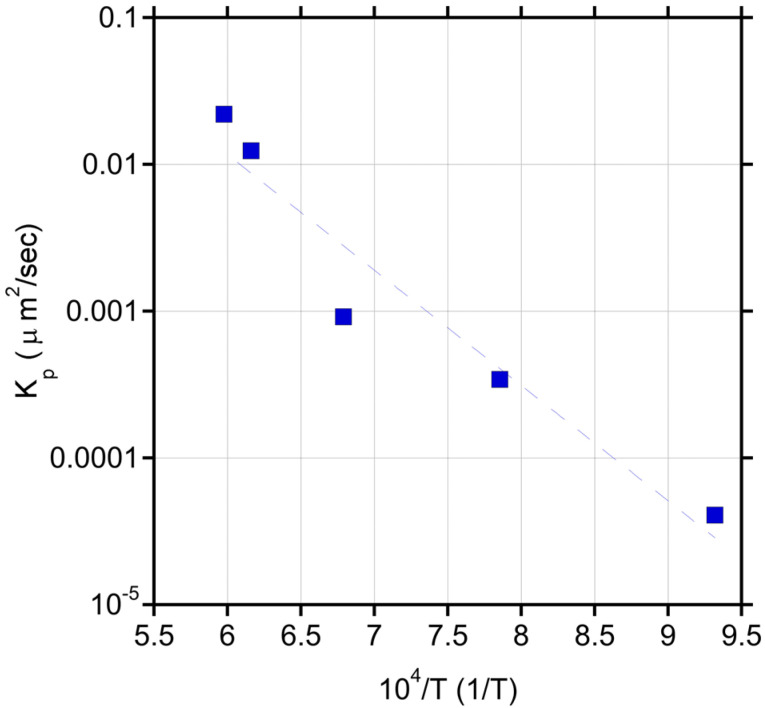
K_p_ versus 1/T based on the data from Figure 3.

Table 2 shows the resulting *K_p_* and A values obtained from the data shown in Figure 4. An Arrhenius relationship is established for *K_p_* versus 1/T as shown in Figure 5. From this relationship the following equation is established:

K_p_ (in μm^2^/s) = 609.35 exp(−1.505 × 10^5^/RT)) (2)

where R is the gas constant equal to 8.314 J·mol^−1^·K^−1^. 

**Figure 6:**
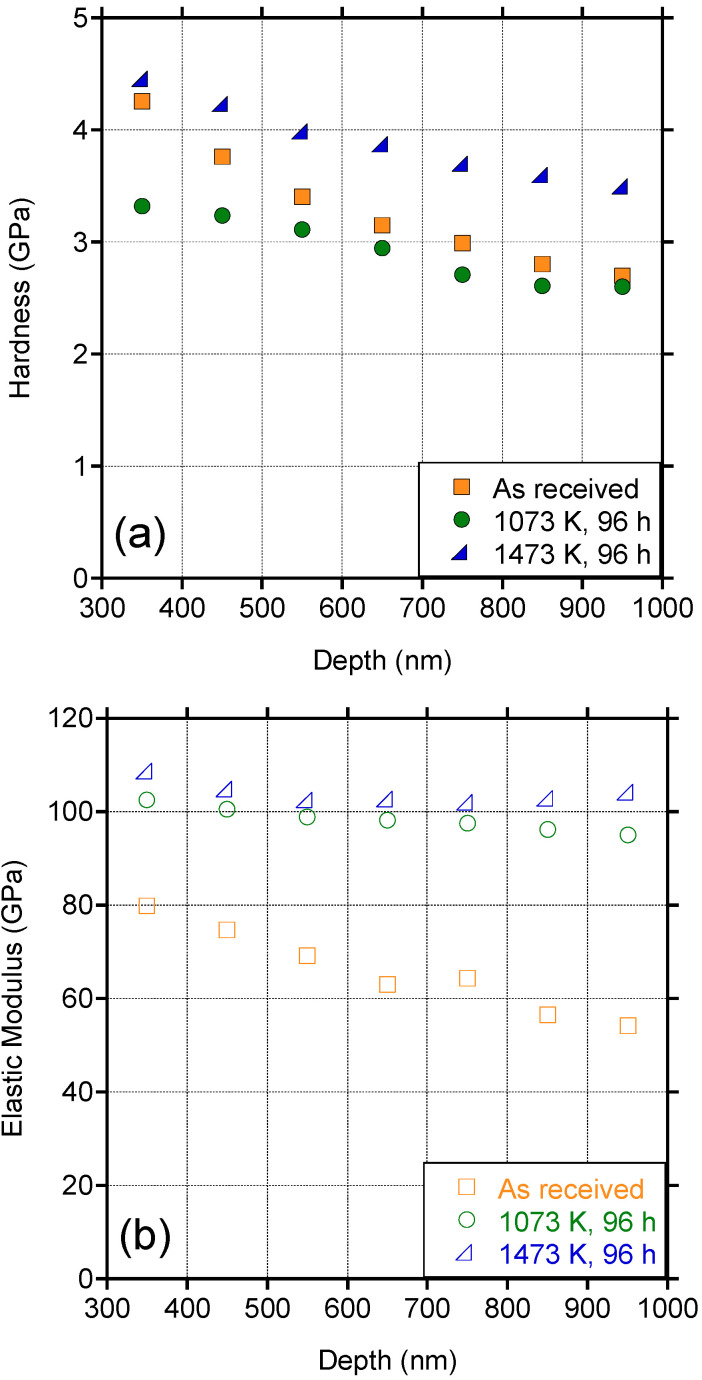
(**a**) Hardness and (**b**) elastic modulus of three Zry-4 samples as a function of depth penetration.

Nanoindentation performed at room temperature was used to determine the hardness and reduced modulus, E_r_ (GPa), of the as-received and carburized Zry-4 samples through solid-state reaction at 1073 K and 1473 K for 96 h. The reduced modulus was converted to Young’s modulus E (GPa) according to the following equation:

E = (1 − v^2^)(1/E_r_ − (1 − v_i_^2^)/E_i_)^−1^ (3)

where E_i_ (GPa) and n_i_ are the indenter Young’s modulus and Poisson’s ratio, respectively, and n is the sample Poisson’s ratio. The hardness H (GPa) was calculated according to Equation (4):

H = F_max_/A (4)

where F_max_ is the maximum load and A is the indentation area. Figure 6 shows the dependence on Young’s modulus and the hardness of Zry-4 as a function of the penetration depth. The average hardness and elastic modulus of as received Zry-4 alloy are 4.17 ± 0.18 GPa and 79.84 ± 2.8 GPa, respectively. The average hardness in the Zry-4 substrates after carburization at 1073 K and 1473 K for 96 hours did not deviate significantly with values of 3.29 ± 0.24 GPa and 4.40 ± 0.46 GPa, respectively. The Young’s modulus did increase for the carburized samples but no differences were observed between the 1073 K (101.04 ± 2.01 GPa) and the 1473 K (106.18 ± 6.37 GPa) sample. A summary of these results, along with comparison values for Zry-4 from the literature, are shown in Table 3.

## 4. Discussion

The observation and characterization of ZrC growth on Zry-4 have important technological implications for the application of this well-established, neutron-friendly cladding material in SMRs. While the growth of ZrC was expected, the kinetics for its growth have not previously been explored in the literature. In this work, we have established the kinetics of ZrC growth for the temperature range of 1073–1673 K. In almost all cases a ZrC layer grew on the Zry-4 substrate, however for temperatures below 1273 K, this layer was discontinuous and, in many cases, only 1 μm in thickness. This is highlighted by the collected XRD pattern at 1073 K, where ZrC diffraction peaks are absent (Figure 3). ZrC peaks only begin to emerge after a 200 K increase in temperature (with an equivalent carburization time), and at 1473 K, the carbide peaks become dominant. The ZrC grown on the Zry-4 substrates under such conditions is hypo-stoichiometric with the closest match being ZrC_0.63_. This is represented by the shifts in the ZrC peaks in the diffraction patterns when compared with the reference pattern for stoichiometric ZrC and is attributed to the wide homogeneity range of ZrC at elevated temperatures [28]. Moreover, as carburization temperature increases, the strains in the carbide coating layer also increase, which is indicated by the ZrC peak broadening in Figure 3.

Microstructural evaluations also confirmed that below 1273 K, exposure to graphite for at least 48 h was necessary to form a semi-continuous ZrC layer. A marked increase in the growth rate for ZrC was seen for temperatures in the range of 1473–1673 K, which were selected to simulate an off-normal scenario during SMR operation. It is noted that while the majority of the testing was through solid-state reaction, pack carburization was also tested for one temperature (1623 K) and it was found that the ZrC that formed at 1623 K was on average thicker, even though less uniform, compared to that which formed at a higher temperature (1673 K) through a solid-state reaction. The difference in response is attributed to the fact that the pack carburization method is expected to be more reactive due to the increased surface area than the solid-state formation which relies on establishing intimate contact throughout the test duration. The Arrhenius relationship established for K_p_ in the range of 1073–1673 K (Equation (2)) provides an activation energy of 150.49 KJ/mol for the formation of ZrC and it is within the order of magnitude of activation energies reported for carbide formation on other transition metal systems [29]. It is also similar to the activation energy for lattice diffusion of C in ZrC at 113.2 KJ/mol as reported in [30]. 

Rapid ZrC growth kinetics between 1073 and 1173 K, the nominal operating temperatures for SMRs, would be a disadvantage for this material as it could mean that the full thickness of a Zry-4 cladding tube could be converted to ZrC during the reactor’s lifetime. Due to the fact that ZrC is a brittle ceramic, the tube would then feature a significant reduction in mechanical integrity, thus, compromising safe operation. Our results indicate that for the operational temperatures of interest, the carburization of Zry-4 due to contact with graphite does not pose a threat to the mechanical integrity of the cladding. As an example, after three years of operation at 1073 K, a 60 μm thick ZrC layer would have developed on Zry-4 cladding. Assuming a 600 μm thickness [31], typical in LWR reactors, this means that only 10% of the overall thickness would have carburized. For an operational temperature of 1273 K, these numbers would increase to 177 μm for the carbide thickness and 30% for the overall carburized thickness. 

In fact, the formation of a thin refractory ZrC layer could serve as an environmental barrier for the fuel-cladding assembly in high-temperature accident scenarios and enhance the fission retention capability of the Zry-4 cladding. ZrC has been considered for many years as a protective coating to replace SiC in Triso-coated fuel particles as it has been proven to have good fission product retention characteristics, especially for caesium and palladium [32,33]. The ZrC layer can also act as a buffer layer against further C diffusion for applications that require extended operating lifetimes. Since ZrC can be hypo-stoichiometric at reactor temperatures, incorporation of free C from the graphite core-block in the carbide layer would limit further C ingress into the fuel-cladding assembly. This concept has been demonstrated for limiting the deleterious long-term carburization effects of carbide-based nuclear fuels [34,35]. This, in addition to evidence from our nanoindentation results that there is minimal impact on the mechanical properties (hardness and Young’s modulus) of the Zry-4 substrate after carburization for 96 h point to a robust cladding option that could remain structurally sound under typical SMR operating conditions. In this work we have established the mechanical properties of the carburized substrates after 96 h of exposure to graphite, however, longer-length testing needs to be performed to fully assess how the mechanical properties will be affected during in-reactor operation. If the mechanical integrity of the Zr-based cladding is established for long-term operation (i.e., 3 years) then this could eliminate the need to use cladding materials that are less favorable from a neutronic perspective such as Fe-based alloys, as shown in Table 1.

## 5. Conclusions

In summary, the kinetics of ZrC growth on Zry-4 due to reaction with graphite were assessed over the temperature range of 1073–1673 K through solid-state reaction and pack carburization. The following conclusions were established:

- In all cases a parabolic growth was established.
- Assessments of the resulting ZrC thicknesses as a function of time and temperature led to the development of an equation for parabolic constant K_p_ as a function of temperature.
- In-situ ZrC growth was shown to be sluggish at nominal SMR operating temperatures (e.g., below 1173 K) and, therefore, it is not expected that the ZrC layer will impact the thermal conductivity or the mechanical integrity of the cladding.
- XRD results revealed a sub-stoichiometric ZrC layer, which in reactor operation could act as a sink for C diffusion, limiting the ingress of C in the Zry-4 bulk.
- Carburized Zry-4 substrates (96 h at 1073 K and 1473 K) showed no significant changes in the hardness or Young’s modulus values as compared to the as-received alloy.

The results presented here confirm that Zry-4 could be used as a cladding material in SMR designs where a graphite core-block is utilized, thus, contributing to the overall neutronic efficiency of the reactor and promoting enhanced commercial viability and performance.

## Figures and Tables

**Table 1:** Summary of key thermomechanical properties for Zry-4 and an alternative Fe-based alloy for LWR cladding applications, APMT.

Property	Zry-4	FeCrAl (Kanthal APMT)
Thermal conductivity (W/m·K)	11.96 [12]	10.78 [13]
Melting temperature (K)	2033 [14]	1773 [15]
Neutron absorption cross-section [16] (barns)	0.185	2.55
Elastic modulus (GPa)	99–115 [17]	184.4 [13]
Hardness (GPa)	2–3 [17]	2.66–3.01 [18]

**Table 2:** K_p_ and A values as a function of temperature based on the data presented in Figure 3.

	1073 K	1273 K	1473 K	1623 K	1673 K
$K_{p}$ (μm^2^/h)	0.1467	1.2372	3.2956	44.6798	79.2758
A (μm)	−0.4827	−2.4193	−1.1856	−3.3684	−18.546

**Table 3:** Summary of elastic modulus and hardness values as obtained from nanoindentation tests on as received Zry-4 and carburized Zry-4.

Material	Elastic Modulus (GPa)	Hardness (GPa)
Zry-4	79.84 ± 2.8	4.17 ± 0.18
Zry-4 [17]	99–115	2–3
Zry-4 [26]	94–96	-
Zry-4 [27]	-	3
Zry-4 (carburized 1073 K, 96 h)	101.04 ± 2.01	3.19 ± 0.24
Zry-4 (carburized 1473 K, 96 h)	106.18 ± 6.37	4.40 ± 0.46

## Data Availability

Not applicable.
